# Three recommendations to the new Director-General of the WHO on how to deliver for girls and women

**DOI:** 10.1186/s12978-017-0346-x

**Published:** 2017-07-14

**Authors:** Katja Iversen

**Affiliations:** Women Deliver, 588 Broadway, Suite 905, New York, NY 10012 USA

## Plain English summary

In May, Dr. Tedros Adhanom Ghebreyesus – Dr. Tedros, as he is commonly referred to – was elected the next Director-General of the World Health Organization (WHO) [1].It was a historic moment for two reasons. It was the first-ever formal Director-General election by all government Member States of the WHO and Dr. Tedros will be the first Director-General of the WHO to hail from Africa [2, 3].

Dr. Tedros, the former Minister of Health of Ethiopia, who saw his country through a bold scale-up of the health system including an impressive Health Extension Worker Program, has quite the job in front of him [1]. The WHO carries an ambitious mandate: to ‘ensure the highest attainable level of health for all people’ [4].Within this expansive mandate comes nuance – disease specific and context specific distinctions layered with various structural, social, and environmental determinants – and competing priorities. Add to that a loud cry for reform of the WHO from governments across the globe, and the new Director-General surely has his work cut out for him.

Early performance of a chief executive is often measured against their achievement through the first 100 days, and Dr. Tedros will be under scrutiny from many sides. However, the WHO is a complex, Member State-led organization and changing it will take time. Dr. Tedros should get a little more than 100 days to turn the ship around – and he should get the support he needs from the community and many stakeholders both within and outside the health space.

Women Deliver, as a recognized global advocacy organization, commits to supporting and helping where we can – and also to holding him accountable to his promises, and nudging when and if needed [5]. That is what friends are for.

Here are three recommendations that Dr. Tedros should focus on during his first three months – and beyond:

First, advancing gender equality and investing in the health, rights, and wellbeing of girls and women, is an investment that multiplies. All evidence shows that investing in women is an investment in progress for all – families, communities, and entire countries. It is essential to the realization of all the Sustainable Development Goals (SDGs), especially SDG 3, which is devoted to the health and wellbeing of all, at all stages in life [6]. So, an absolute first for Dr. Tedros is to continue to stand up for investments in women’s health and wellbeing, including women’s sexual and reproductive health and rights, and to apply a gender lens to noncommunicable diseases. Simultaneously, he should ensure a much better gender balance across the organization, including the advancement of the WHO’s new policy on gender equality in staffing. Adopted early in 2017, this policy calls for at least a 1.5% increase annually for the next five years in the number of female staff members at the P4 level and above in WHO professional postings, with the goal of gender parity at the highest levels [7]. But his leadership must go beyond the walls of the WHO to speak up against the current attacks on women’s ability to decide on their own fertility, including access to modern contraception and abortion. He should work closely with civil society organizations to ensure that evidence-based solutions from the WHO are translated into standard guidelines, practices, and policies that will save the lives of women, children, and adolescents everywhere. Dr. Tedros’ key priority – Universal Health Coverage – must not compromise on the provision of sexual and reproductive health for all. It must be an essential part of services and coverage [5].

Second, it is a popular and true saying that if we focus on health and health alone, we will never solve the health problems the world faces. To make the advancements in health that are currently envisioned across global strategies and plans, the WHO cannot afford to focus on health interventions alone. From the SDGs to the Global Strategy on Women’s, Children’s, and Adolescents’ Health to The Global Strategy on Human Resources for Health: Workforce 2030, the current directive to fuel progress is immense and cross-issue in nature. The SDGs represent an interconnected and indivisible vision and roadmap [8, 9]. To sustainably improve health and wellbeing, the WHO must also work with sister UN agencies, governments, and other organizations to further an integrated approach. This includes enhancing girls’ and women’s access to education, strengthening their access to clean water and sanitation, eliminating gender-based violence, and advancing their economic opportunities. Simply put, Dr. Tedros should seize upon reciprocal development relationships; for example, educational attainment is as essential to good health as good health is to educational attainment – and a key component in having a good health workforce. To reach the desired progress in global health, Dr. Tedros must take an integrated approach together with partners working on gender equality, education, economic development, and so on, because to fuel healthy communities, you have to look at the whole person – not just a disease or a body part. To pursue this new mode of integrated development effectively, Dr. Tedros must recognize the central role of civil society engagement, advocacy, and policy efforts designed to bridge the silos of development to yield coherent strategies that get the political attention and funding they deserve to achieve the SDGs.

A final recommendation that will yield results is to address the health, rights, wellbeing, and involvement of young people, including adolescents, in the work. Young people between the ages of 10–24 represent a quarter of the world’s population^,^ and within 48 of the world’s least developed countries, half of the population are under 18 [10]. Recognizing and responding to the growing demographic of young people and adolescents and meeting their aspirations and needs, not least sexual and reproductive health needs, of this rapidly growing population group will define progress made not only in health, but also in development writ large. Young people and adolescents need access to information, resources, and services to be able to make sound decisions about their health and wellbeing. Young people must also have a seat at the table and be involved in program planning, implementation, and decision-making processes that affect their lives. This means youth and adolescent groups need to be integrated into all civil society and othe﻿r engagements that the WHO undertakes. Women Deliver recommends that Dr. Tedros prioritize young people’s and adolescents’ health, rights, and wellbeing in the WHO’s work, and add a Youth Advisory Board to his team. Women Deliver stands ready to support Dr. Tedros in this and will help establish it by adhering to strong guidelines of meaningful youth engagement and by recommending some of the wonderful and knowledgeable young people from across the globe, whom we work with every day.

Only time will tell if Dr. Tedros is able to deliver on the seemingly herculean mandate of his new role. We, at Women Deliver, are optimistic, supportive, and will be doing our part to help ensure his success. The chances will definitely be better if he takes on board our recommendations and delivers for girls and women.
